# Providing a toolbox for genomic engineering of *Trichoderma aggressivum*

**DOI:** 10.1128/spectrum.00966-25

**Published:** 2025-07-31

**Authors:** Matthias Schmal, Lara T. S. Kramer, Robert L. Mach, Astrid R. Mach-Aigner, Christian Zimmermann

**Affiliations:** 1 Institute of Chemical, Environmental and Bioscience Engineering, TU Wien 27259, Vienna, Austria; University of Natural Resources and Life Sciences Vienna, Vienna, Austria

**Keywords:** *Trichoderma*, genome editing, transformation, marker, auxotrophy

## Abstract

Fungi belonging to the genus of *Trichoderma* have a long history of infecting crops of edible fungi and reducing the yield. *Trichoderma aggressivum* is the main causal agent of green mold disease in *Agaricus bisporus*. Despite its infamous role as a persistent and aggressive contamination in *A. bisporus* crops, *T. aggressivum* can also be used as a biocontrol agent or as a promoter of plant growth. In order to work efficiently with *T. aggressivum* on a molecular biology level, a transformation protocol is required. This study provides a detailed protocol on how to perform a transformation in *T. aggressivum* using plasmid DNA for ectopic integration. In addition, a Cas9-RNP-based approach has been established for genome editing. We performed two transformations to confirm the usability of the *hph* gene as well as the *pyr4* gene from *Trichoderma reesei* as selection markers. First, we integrated the *hph* gene ectopically and determined the overall transformation efficiency. In the second transformation experiment, we disrupted the ornithine-5′ phosphate decarboxylase gene of *T. aggressivum* by using the CRISPR-Cas9 approach. In total, four candidates showed the expected uridine auxotrophy and resistance to 5-FOA. Additionally, the genomic locus around the CRISPR target sites was sequenced to determine the exact circumstances around the gene disruption. To complete the genetic toolbox for *T. aggressivum*, the *pyr4* gene of *T. reesei* was tested as a suitable selection marker in one of the generated uridine auxotrophic strains.

**IMPORTANCE:**

Researchers need an efficient tool for genomic manipulation to investigate the fundamental biology of mycoparasitism of *T. aggressivum* and its correlation to secondary metabolites. We provide a protocol for transformation of *T. aggressivum* and successfully demonstrated transformation of *T. aggressivum* using a plasmid and genome editing applying a Cas9-RNP-based strategy. Simultaneously, we established two selection markers, the *hph* gene and *pyr4* gene from *T. reesei*. By applying these methods, we give researchers the tools needed to investigate *T. aggressivum* on a deeper level. Possible applications include activation of biosynthetic gene clusters of secondary metabolites to determine the biosynthetic pathway and biotechnological applications of these compounds.

## INTRODUCTION

Among the most cultivated edible fungi are *Pleurotus ostreatus* (oyster mushrooms), *Lentinula edodes* (shiitake), and *Agaricus bisporus* (cultivated mushroom). The genus *Trichoderma* is known to be the cause of mold in these edible fungi farms and negatively influence the yield of fungi crops (1–3). The cause of green mold in *A. bisporus* cultures was first described in 1986 as *Trichoderma harzianum* during an epidemic in the United Kingdom (3) and then later reclassified as *Trichoderma aggressivum* in 2002 (4). *T. aggressivum* was then isolated as a mycoparasite of *A. bisporus* cultures around the world: North America (5), Spain (6), Hungary (7), and Turkey (8), to name a few countries.

However, *Trichoderma* spp. are not exclusively known as mycoparasites. In the early 1930s, the mycoparasitic properties of *Trichoderma viride* against plant pathogens were discovered (9). Since then, many more applications of *Trichoderma* spp. as biocontrol agents have been reported (10). These applications are not limited to fungi but extend to phytoparasitic nematodes (11). In addition, *Trichoderma* spp. can have a positive impact on plant growth through the secretion of various compounds (12, 13). Such beneficial effects in plant cultivation have also been shown for *T. aggressivum*. The use of *T. aggressivum* as a biocontrol agent against other fungi (14) as well as the ability to promote plant growth (15) was recently shown.

Many of the described applications and the fundamental biology of its mycoparasitism towards *A. bisporus* can be related to secondary metabolites, be it the antifungal activity necessary to act as a biocontrol agent (16) or the stimulating effect of plant growth (15). Therefore, a deeper understanding of the entire range of secondary metabolites produced by *T. aggressivum* is of great interest. Usually, secondary metabolites are not produced under laboratory conditions. The ability to manipulate *T. aggressivum* on a genetic level allows researchers to activate these biosynthetic gene clusters (17). For an in-depth investigation of the biosynthetic pathway, the deletion of single genes is often required, which further underlines the need for a transformation method in *T. aggressivum*. In addition, the great economic role of *T. aggressivum* in cultivations of *A. bisporus* has led researchers to look closely into the interplay of these two fungi. However, the fundamental biology and the gene regulation involved in this interplay have not been investigated in great depth, another field of research where a transformation protocol is useful.

Many transformation protocols for filamentous fungi have been published. In general, they can be split into three major groups: polyethylene glycol (PEG)-mediated transformation of protoplasts, *Agrobacterium tumefaciens*-mediated transformation, and other methods, such as electroporation, lipofection, or biolistic transformation, which are used if the first two transformation protocols do not work (18–20). Each method comes with its advantages and disadvantages. PEG-mediated transformations are straightforward to perform and generally yield high number of transformants. However, generating viable protoplasts is generally the limiting factor. In addition, fungal cell walls are diverse and each strain needs an optimized protoplastation protocol. If no protoplasts can be generated, *Agrobacterium*-mediated transformations can also produce a large number of transformants, but the transformation efficiency depends on the particular *A. tumefaciens* strain used (21). The discovery of CRISPR-Cas9 for genome editing gave researchers a powerful tool to induce double-strand breaks with high precision (22). The Cas9 enzyme can be delivered in three ways: integrated in the genome, on a plasmid, or together with the single guide RNA as a RNP complex (23). Furthermore, to increase the number of recombination events, the organism should be non-homologous end joining (NHEJ)-deficient (24).

Here, we describe a method that enables researchers to efficiently transform *T. aggressivum* and disrupt genes based on double-strand breaks in the DNA induced by CRISPR-Cas9 using an in vitro assembled Cas9-RNP complex. By delivering a Cas9-RNP into the cells instead of expressing Cas9 from the genome or a plasmid, the transformation efficiency is increased and off-target effects are minimized (25–29).

In this study, the Cas9-RNP-based approach has been tested by disruption of the *pyr4* gene encoding for ornithine-5′ phosphate decarboxylase. This resulted in an uridine auxotrophic strain. Furthermore, the *pyr4* gene from *T. reesei* was established as a selection marker for genetic manipulations. In addition, we demonstrated the applicability of the *hph* gene as a genetic marker in *T. aggressivum*. The *hph* gene originates from *Escherichia coli* and phosphorylates the antibiotic Hygromycin B, thus inactivating it.

## MATERIALS AND METHODS

### Generation of protoplasts

The protoplastation protocol is adapted from current methods used for transformation of *Trichoderma reesei* (30). *Trichoderma aggressivum* f. *europaeum* CBS100526 (4) was cultivated on malt extract plates (MEX) containing 3% malt extract (Merck, Darmstadt, Germany), 0.1% Peptone (Merck, Darmstadt, Germany) and 1.5% agar (VWR, Radnor, PA, USA) for approximately one week at 30°C in darkness. Fresh spores were suspended in 2 mL of a physiological NaCl (VWR, Radnor, PA, USA) solution containing 0.05% Tween 80 (Merck, Darmstadt, Germany) until the solution turned medium green (OD_600_ of approx. 5). 1.5 mL of the spore suspension was added to 50 mL MEX medium and incubated at 30°C and 200 rpm in an incubator shaker for 18 h. The cells are then retained in a 0.22 µm steritop filter (Merck, Darmstadt, Germany) and washed with 200 mL sterile dH_2_O. The mycelium is resuspended in the protoplasting solution (2 g Vinotaste Pro [Novozymes, Bagsvæ rd, Denmark] and 0.03 g Proteinase K [Roth, Karlsruhe, Germany] in 30 mL of Buffer A [100 mM KH2PO4 {Merck, Darmstadt, Germany}, 1.2 M sorbitol {Sigma-Aldrich, St. Louis, MO, USA}, pH adjusted to 5.6 using KOH], filtered sterile using a 45 µm filter [Roth, Karlsruhe, Germany]) and put into a fresh sterile 100 mL Erlenmeyer flask. The cell suspension was incubated for 3–4 h at 30°C and 120 rpm, until protoplasts were visible under the light microscope. To remove the remaining patches of mycelium, the protoplast solution was filtered through two layers of Miracloth (Merck, Darmstadt, Germany) into a sterile 50 mL centrifuge tubes and filled up to 40 mL with ice-cold 1.2 M sorbitol. The protoplast suspension was centrifuged for 5 min with 1,500×*g* at 4°C. The supernatant was carefully discarded and another 30 mL of ice-cold 1.2 M sorbitol was added. The centrifugation was repeated with 1,500×*g*. Again, the supernatant was discarded and the cell pellet was resuspended in 1 mL Buffer B (1 M sorbitol, 10 mM Tris-HCl [Merck, Darmstadt, Germany] pH 7.5, 25 mM CaCl_2_ [Sigma-Aldrich, St. Louis, MO, USA]). At this point, protoplasts were counted using a Thoma chamber.

### Transformation using a plasmid as donor DNA

The transformation process starts by gently mixing 15 µg DNA in 150 µL Buffer B, 100 µL ice-cold 20% PEG (mix 1 part of 60% PEG [w/v% PEG4000 {Sigma-Aldrich, St. Louis, MO, USA}, 10 mM Tris-HCl pH 7.5, 10 mM CaCl_2_] with 2 parts Buffer B) and 100 µL of protoplast suspension. The mixture is incubated on ice for 30 min. Subsequently, 50, 200, and 500 µL 60% PEG is added stepwise. After each addition of 60% PEG, the cells are mixed gently by rolling the tube in a tilted position. The cells were incubated at room temperature for 20 min. Then, Buffer C (1 M sorbitol, 10 mM Tris-HCl pH 7.5) was added in steps of 200 µL, 400 µL, 1 mL, and 2.5 mL with gentle mixing between each step. Finally, the whole mix is filled up to 50 mL with warm selection media containing in addition 1 M sucrose (Wiener Zucker, Agrana, Vienna, Austria) and 1.5% agar and poured quickly into a petri dish with 10-cm diameter (Greiner Bio-One, Kremsmünster, Austria). The plates were incubated at 30°C under light until colonies were visible. Selection medium was either MEX medium containing 250 µL/L Hygromycin B (product number 400051, Sigma-Aldrich, St. Louis, MO, USA), or modified Mandels Andreotti (MA) medium (31) (2 g KH2PO4, 1.4 g (NH4)2SO4, 0.3 g Urea, 0.005 g FeSO4.7H2O, 0.0016 g MnSO4.H2O, 0.0014 g ZnSO4.7H2O, 0.002 g CoCl2, 0.3 g MgSO4.7H2O, 0.3 g CaCl2, 1% [w/v] glucose) supplemented with 5 mM uridine (Roth, Karlsruhe, Germany) or 1 g L^-1^ 5-fluoroorotic acid (5-FOA) (abcr, Karlsruhe, Germany) if required. The transformation efficiency t_*eff*_ was calculated according to equation 1.


(1) $$t_{eff}=\frac{\text{ number of colonies }}{\mu g\;\text{DNA}}$$

### Transformation using a Cas9-RNP-based system with guide RNAs

The online tool CHOPCHOP was used (32) (https://chopchop.cbu.uib.no/) to scan for potential target sites in the region of interest. To find potential off-targets, a BLAST search was conducted. The online tool EnGen sgRNA Designer (https://sgrna.neb.com/#!/sgrna) from NEB was used to design target-specific oligos. These oligos include a promoter for the T7 RNA polymerase and the sequences required for the interaction with the Cas9 enzyme. RNA synthesis was performed using the EnGen sgRNA Synthesis Kit (E3322, New England Biolabs, Ipswich, MA, USA). Subsequent purification of RNA was done with the Monarch RNA Cleanup Kit (T2040, New England Biolabs, Ipswich, MA, USA). RNAs were refolded by heating the required amount of RNA to 85°C for 1 min and subsequently cooled down to 4°C at a rate of 0.1°C s^-1^. The transformation starts by mixing 15 µL 10× Cas9-Buffer (0.467 g HEPES [Roth, Karlsruhe, Germany], 1.118 g KCl [Merck, Darmstadt, Germany], 0.203 g MgSO4 . 7 H2O, 40 µL 0.25M EDTA [VWR, Radnor, PA, USA] dissolved in 80 mL ddH2O, pH set to 7.5 with KOH, 50 µL 1 M DTT [Sigma-Aldrich, St. Louis, MO, USA], filled up to 100 mL) with 4.25 µL EnGen Spy Cas9 HF1 (M0677, New England Biolabs, Ipswich, MA, USA) and a total of 2.7 µg of sgRNA (1.35 µg each in the case of two different sgRNAs). The mixture is filled up to 150 µL with Buffer B and then incubated at 37°C for 10 min. After adding 100 µL 20% PEG and 100 µL protoplast suspension, the transformation follows the same protocol as for the transformation using a plasmid.

### Homokaryon selection of candidates

Colonies are usually visible 2–3 days after transformation. They were cut out from the agar and transferred to fresh selection media plates without sucrose and incubated at 30°C until green spores were clearly visible. For homokaryon selection, they were streaked on selection media containing 0.1% (w/v) IGEPAL (Sigma-Aldrich, St. Louis, MO, USA). Single colonies were transferred to fresh agar plates and incubated at 30°C until sporulation occurred.

### DNA extraction and genotyping

Approximately 100 mg of fresh mycelium was pressed gently between Whatman filter paper to remove excess medium and placed in 2 mL cryo vials. Glass beads were added to the mycelium: one 5-mm bead, 0.25 g of 1 mm and 0.37 g of 0.1 mm sized. After adding 1 mL of CTAB buffer (1.4 M NaCl, 100 mM Tris-HCl pH 8.0, 10 mM EDTA, 2% [w/v] CTAB [Merck, Darmstadt, Germany], 1% [w/v] polyvinylpyrrolidone [Sigma-Aldrich, St. Louis, MO, USA]) cells were lysed with the help of a FastPrep (MP Biomedicals, Santa Ana, CA, USA) on the following settings: 6 m s^-1^ for 30 s. Subsequently, the vials were incubated at 65 °C for 20 min. Then, 800 µL supernatant was transferred into a 2-mL reaction tube. First, 400 µL phenol (Applichem, Darmstadt, Germany) was added and the mixture was shaken vigorously in a horizontal position for 30 s. Then, 400 µL chloroform (Sigma-Aldrich, St. Louis, MO, USA) was added, shaken in a horizontal position, and incubated at RT for 10 min. After centrifugation for 10 min with 12,000×*g* at 4°C, 650 µL of the aqueous phase was transferred into a fresh 1.5-mL reaction tube and mixed with an equal amount of chloroform. Again, the mixture was centrifuged like before, and 550 µL of the aqueous phase was transferred. The previous step is repeated, and 500 µL of the aqueous phase is transferred into a fresh tube. To remove RNA from the sample, 1.5 µL RNase A (10 mg/mL, Thermo Fisher Scientific, Waltham, MA, USA) was added and incubated at 37°C for 30 min. After the addition of 350 µL isopropanol (Merck, Darmstadt, Germany), the mixture was inverted six times, incubated at RT for 10 min, and then centrifuged with 21,000×*g* at 4°C for 30 min. The supernatant was removed carefully, and 1 mL of ice-cold 70% ethanol was added. The centrifugation was repeated for 10 min, and the supernatant was removed again. The DNA was then air-dried at 55°C and resuspended in 100 µL dH2O. Concentration was determined using a NanoDrop One (Thermo Fisher Scientific, Waltham, MA, USA). To check the deletion of the *pyr4* gene of *T. aggressivum,* the Q5 Hi-Fi polymerase (M0491, New England Biolabs, Ipswich, MA, USA) was used. Primers pyr4-fwd and pyr4-rev were used to amplify the *pyr4* locus. For sequencing, the primer pyr4-seq was used. The location of the primers is indicated in Fig. 2A. To confirm the integration of the *pyr4* gene of *T. reesei* into the genome of *T. aggressivum*, a PCR was performed using the Q5 Hi-Fi polymerase and primer that are specific for *pyr4* of *T. reesei*. All primer sequences are stated in Table S1.

## RESULTS AND DISCUSSION

### Generation of protoplasts and transformation of *T. aggressivum* using the *hph* gene as selection marker

**Fig 1:**
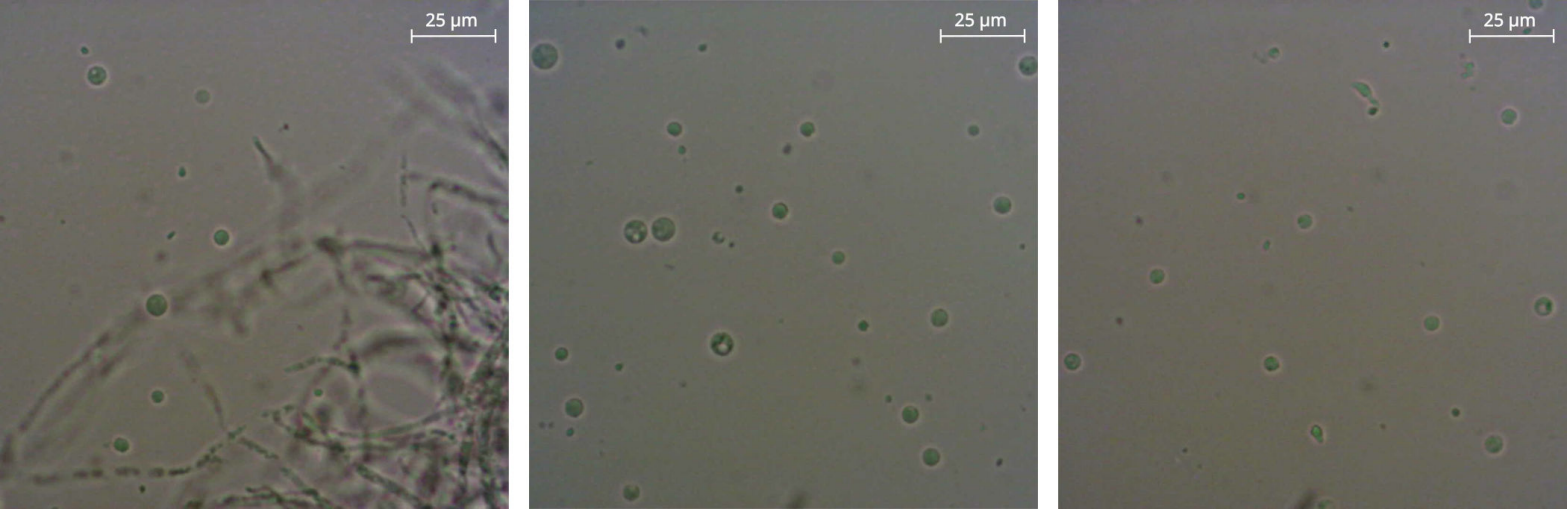
Light microscopic images of typical protoplasts after incubation in protoplasting solution for 3 h.

For successful transformations, a reproducible way of generating protoplasts is a crucial step. Several enzyme mixtures were tested, and Vinotaste Pro in combination with Proteinase K worked best (see Materials and Methods for details). After 3 h of incubation with the protoplasting solution, the amount and quality of the protoplasts were verified using a light microscope. Figure 1 shows representative images of a successful process. Most cells show a typical round shape. However, patches of mycelium are still present after 3 h of incubation. After filtration, the cells are ready to be used further for transformation.

To test the applicability of the *hph* gene as a selection marker, *T. aggressivum* was transformed with the plasmid pAN7-1 (33). The protoplasts were counted (382,000 cells mL^-1^) and then used for transformation using different amounts of total DNA of pAN7-1: 2.5 µg, 5 µg, and 10 µg. The transformation was performed in biological triplicates. The mean CFUs for each concentration of the plasmid are 79, 143, and 197 in ascending order of the plasmid concentration. This results in an overall transformation efficiency of t_*eff*_ = 26.63 cfu µg^-1^ (see equation 1).

### Deletion of *pyr4* resulted in uridine auxotrophy

**Fig 2:**
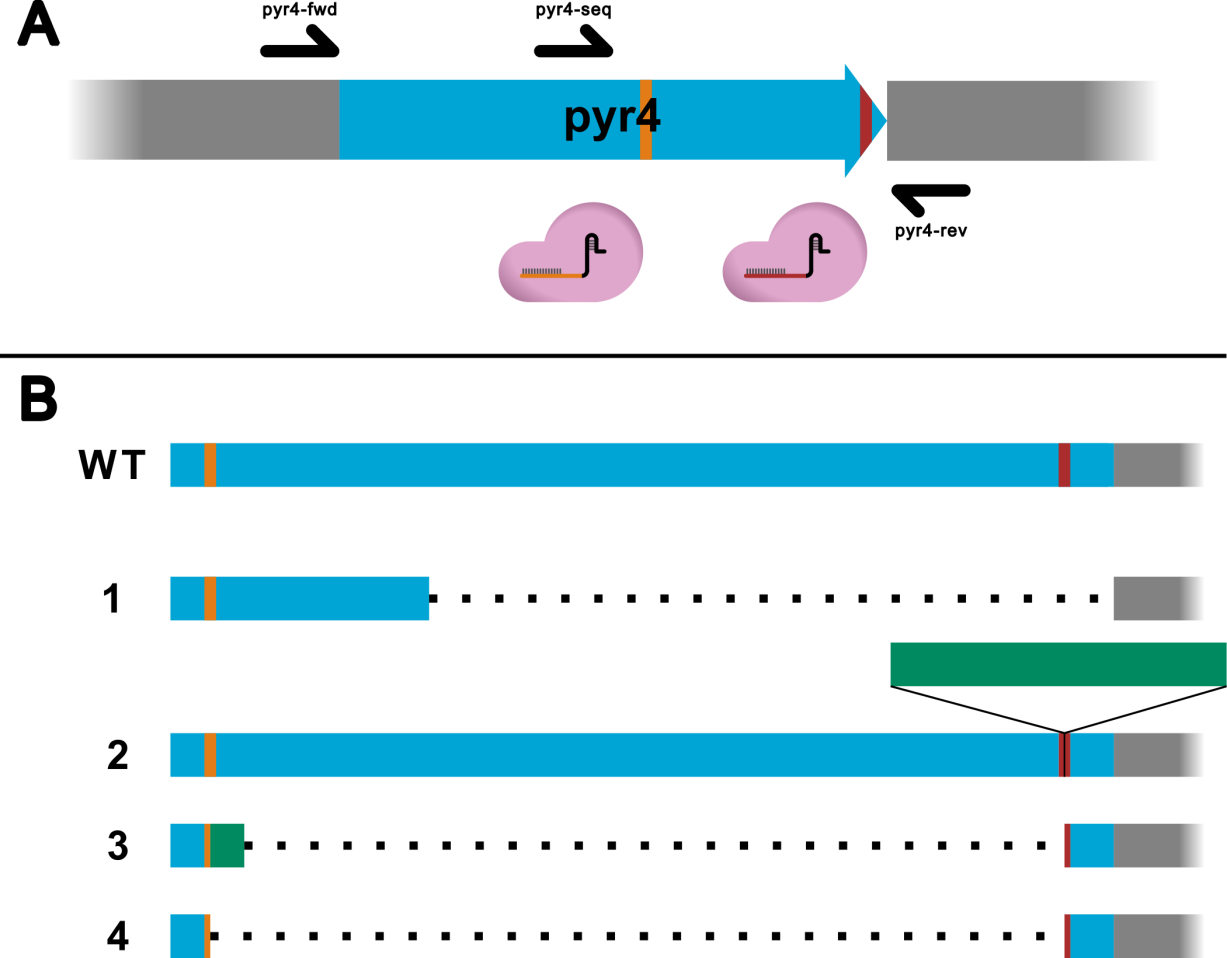
(A) The experimental setup for the gene disruption of *pyr4*. The blue arrow represents the *pyr4* gene of *T. aggressivum*, gray boxes represent adjacent genomic sequences. Marked in orange and red are the locations of the CRISPR target sequences. Black arrows indicate primer binding sites. (B) A graphical representation of the sequence alignments of the target locus of obtained candidate strains against the wild-type locus (WT). Marked in blue is the *pyr4* gene of *T. aggressivum*. Gray boxes represent adjacent genomic sequences. CRISPR target sites are marked in orange and red respectively. Inserted or replaced DNA sequences are colored green.

Next, we aimed at deleting the orotidine-5′ phosphate decarboxylase (OMP, encoded by *pyr4*). We decided to use a Cas9-RNP-based approach. Two different guide RNAs were used to induce double-strand breaks in the *pyr4* gene. Figure 2 shows the genome modification strategy and the changes of the target sequence in tested candidate transformed strains. The expected result is a deletion between both CRISPR-Cas9 target sites. The transformation yielded 12 colonies in total. After placing the colonies on fresh selection media plates without sorbitol, only four candidates showed significant growth after 3 days. Each of the candidates underwent homokaryon selection. Subsequent genotyping revealed that all four candidates have a disruption of the *pyr4* gene, albeit a different one in each candidate (see Fig. 2). The primer pyr4-seq was used for Sanger sequencing.

**Fig 3:**
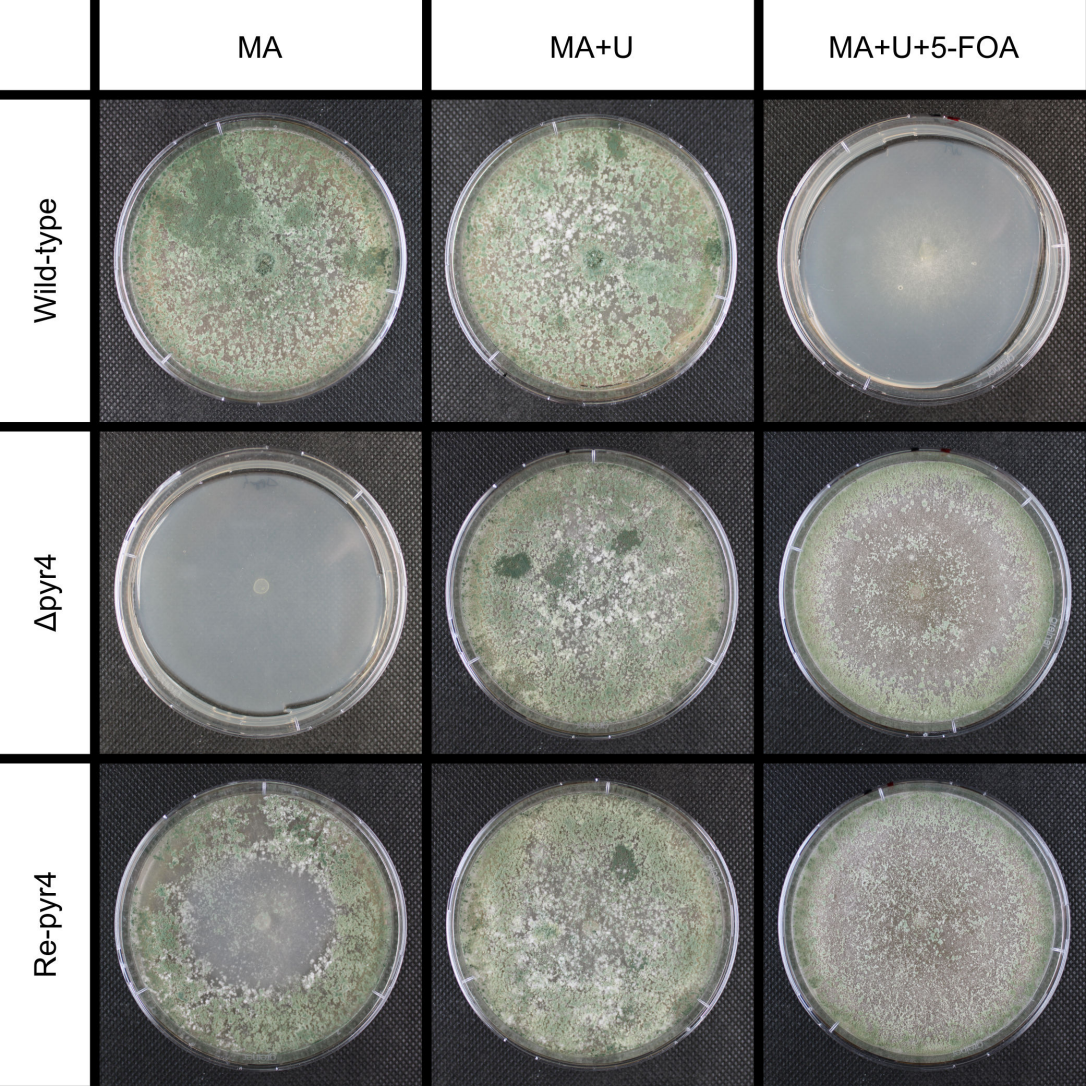
Phenotyping of *T.* aggressivum wild-type, *T. aggressivum Δpyr4,* and *T.* aggressivum Re-*pyr4*. The cultivation was done at 30°C for 13 days on modified MA plates (MA), plates with uridine (MA+U), and plates supplemented with uridine and 5-FOA (MA+U+5-FOA).

Candidate 1 exhibits a partial deletion of the expected sequence and an additional deletion beyond the 3′ CRISPR target site. Candidate 2 has no deletion at all. Instead, mitochondrial DNA with a length of around 220 nucleotides has been inserted at the second CRISPR target site. In candidate 3, the sequence between the target sites was replaced by an unrelated genomic region of *T. aggressivum*. Candidate 4 has the anticipated deletion (see Fig. 2). In addition to the genotypic characterization of the strains, phenotyping was conducted. All candidates were placed on MA, MA+U, MA+U+5-FOA (see Fig. 3). The first two rows of Fig. 3 show the plates after incubation for 7 days at 30°C in darkness. The wild-type strain was able to grow on MA and MA+U, but the addition of 5-FOA inhibited growth. The growth of the Δ*pyr4* strain was dependent on the presence of uridine in the medium but was not affected by 5-FOA.

### The *pyr4* gene of *T. reesei* can be used as a selection marker

Next, we tested whether the prototrophy can be recovered by ectopic integration of the *T. reesei pyr4* gene. As donor DNA, the plasmid pCD-RPyr4 (34) was used. This plasmid contains the *pyr4* gene from *T. reesei*, including its native promoter and terminator. Three independent transformations of candidate 1 with 15 µg of pCD-RPyr4 were performed and resulted in a mean t*_eff_* = 25.4 cfu µg^-1^ . To check for integration of *pyr4* of *T. reesei,* a total of nine colonies from two separate transformations were picked, placed on fresh plates, and subsequently, the genomic DNA was extracted. PCR was performed with primers (see Table S1) reesei-pyr4-fwd and reesei-pyr4-rev for the *pyr4* of *T. reesei*. The resulting gel image can be seen in Fig. S1. The phenotyping of the *Δpyr4* as well as the Re-*pyr4* strain is also in agreement with the genotype. The wild-type strain was able to grow well on MA and MA+U, but poorly on MA+U+5-FOA. The poor growth on MA+U+5-FOA can be explained by the instability of 5-FOA, which led to a delayed onset of growth. The *Δpyr4* strain did grow on both plates supplemented with uridine, regardless of 5-FOA. After 13 days of incubation, the retransformed strain grew well on MA+U and weaker on MA+U+5-FOA as well as MA (see Fig. 3). This indicates that the *pyr4* of *T. reesei* is not able to fully complement the uridine auxotrophy as the Re-*pyr4* strain needed the uridine supplementation to reach the growth rate of the wild-type strain. In addition, 5-FOA has almost no effect on the growth of the Re-*pyr4* strain on the test plates. Importantly, the *pyr4* gene of *T. reesei* can be used as a selection marker as positive transformants can grow on the minimal medium MA in comparison to the parental Δ*pyr4* strain. This result is confirmed by the fact that performing the transformation protocol with a Δ*pyr4* strain without donor DNA does not produce colonies (data not shown). Cultivation of strains that used the *pyr4* gene of *T. reesei* requires additional uridine supplemented in the medium.

### Final remarks and outlook

We established an easy-to-follow and efficient protocol for transformation of *T. aggressivum*. First, we showed the usability of the *hph* gene as a selection marker. We achieved a t_*eff*_ of about 26 cfu µg^-1^ which is between reported transformation efficiencies of up to 2500 cfu µg^-1^ in the model organism *T. reesei* (35) and the less researched *Trichoderma koningiopsis* with a transformation efficiency of about 1 cfu µg^-1^ (19). Then, we employed a CRISPR-Cas9 approach to disrupt the *pyr4* gene of *T. aggressivum*. This resulted in a uridine auxotrophic strain. In addition, we showed that the *pyr4* gene of *T. reesei* can be used as a selection marker in the *T. aggressivum* Δ*pyr4* strain.

The ability to efficiently disrupt and integrate genes enables researchers to investigate biosynthetic gene clusters. This spans the activation of silent clusters in this organism by integration and over-expressing of cluster-specific transcription factors to scrutinizing the biosynthesis of secondary metabolites by individually deleting genes in a biosynthetic gene cluster. In combination with analytical methods such as high-resolution mass spectrometry, researchers will be able to fully characterize the biosynthetic pathways of secondary metabolites, as previously shown in other organisms (30, 36, 37). In addition, researchers are now able to manipulate the genome of *T. aggressivum* to investigate the fundamental biology involved in mycoparasitism of *T. aggressivum*.

## SUPPLEMENTAL MATERIAL

Supplemental material spectrum.00966-25-s0001.pdf Fig. S1, Table S1, and sequences.

ASM does not own the copyrights to Supplemental Material that may be linked to, or accessed through, an article. The authors have granted ASM a non-exclusive, world-wide license to publish the Supplemental Material files. Please contact the corresponding author directly for reuse.
