# Supplementary material for: Providing a toolbox for genomic engineering of Trichoderma aggressivum
Source: Microbiol Spectr. 2025 Jul 31;13(9):e00966-25. doi: 10.1128/spectrum.00966-25 (PMC12403656; doi:10.1128/spectrum.00966-25)
Supplement: Supplemental material — Fig. S1, Table S1, and sequences. [file spectrum.00966-25-s0001.pdf]

Providing a toolbox for genomic engineering of *Trichoderma aggressivum*

Matthias Schmal,[1] Lara T. Kramer,[1] Robert L. Mach,[1] Astrid R. Mach-Aigner,[1] Christian Zimmermann [1*]

[1]Institute of Chemical, Environmental and Bioscience Engineering, TU Wien, Vienna, 1060, Austria

*Address correspondence to Christian Zimmermann,

christian.zimmermann@tuwien.ac.at.

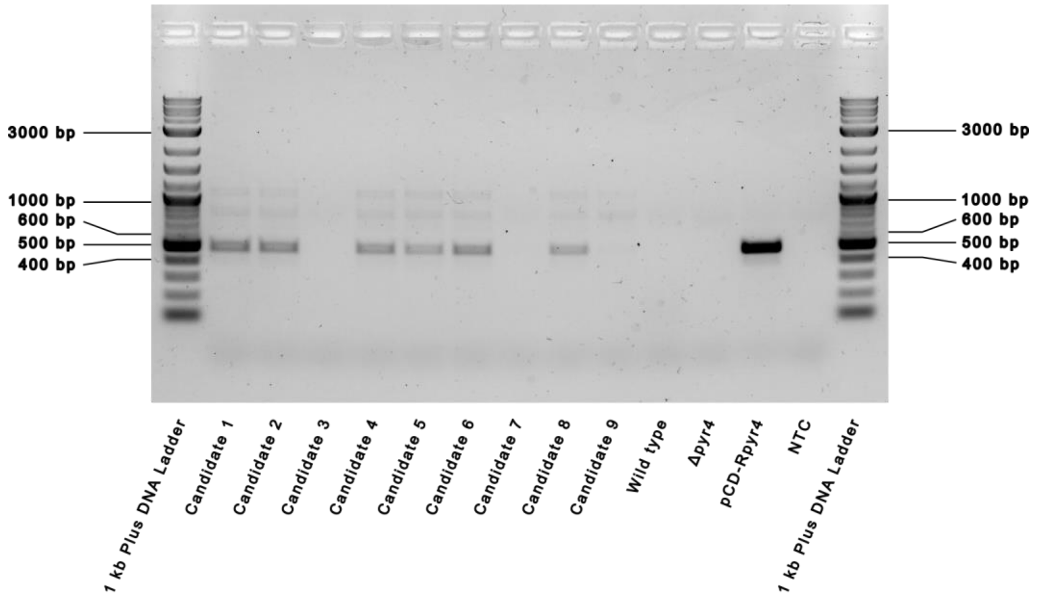

Figure S1: Agarose gel image of Re-pyr4 genotyping. The expected fragment size of the PCR product is 477 bp. Candidates 1, 2, 4, 5, 6 and 8 show a distinct band at the expected size. pCD-Rpyr4 is the positive control and shows a specific band at 477 bp. NTC, no template control.

Table S 1: Oligonucleotide and primer sequences used. Underlined sequences correspond to CRISPR target sites in the *pyr4* gene of *T. aggressivum.*

Name	Sequence (5’-3’)
pyr4-fwd	CGCATTTACGCAGCCATCTT
pyr4-rev	AATGTTCAGTTCGCCTCCAC
pyr4-seq	CATTTACTCTGGCCGAACCG
reesei-pyr4-fwd	GTCAACGGCATGGGCTCCAT
reesei-pyr4-rev	CGATGCCAATCAGCTTGT
Target-specific oligo 1	TTCTAATACGACTCACTATAGGACTCGTACTGCTGGGTGAGTTTTAGAGCTAGA
Target-specific oligo 2	TTCTAATACGACTCACTATAGCCTCGGACCCCGTAGAAGGTTTTAGAGCTAGA

Sequences of the *pyr4* locus of the *T. aggressivum* Δ*pyr4* candidate strains

Candidate 1:

CATTGTTTCCMTCACCACCGTCACCCAGCAGTACGAGTCCGTCGCTTCACCTCGGTTGG
CAAAGGCAATCGCTGAGGGAGACGAGTCACTATTTCCAGGCATTGAGGAGGCACCGCT
AAACCGTGGCCTCTTGATTCTCGCCCAGATGTCTAGCCAAGGCAATTTCATGAACAAGG
AGTACACTGAGGCTTGTGTGGAGGCGAACTGAACATTCTGATAATAATTAATTAAGACGT
CAGAATTCTCGAGGCGGCCGCATGTGCGTCTCCCTATAGTGAGTCGTATTAATTTCGCG
GGCGGAACCCCTATTTGTTTATTTTTCTAAATACATTCAAATATGTATCCGCTCATGAGAC
AATAACCCTGATAAATGCTTCAATAATATTGAAAAAGGAAGAGTATGAGTATTCAACATTT
CCGTGTCGCCCTTATTCCCTTTTTTGCGGCATTTTGCCTTCCTGTTTTTGCTCACCCAGA
AACGCTGGTGAAAGTAAAAGATGCTGAAGATCAGTTGGGTGCACGAGTGGGTTACATC
GAACTGGATCTCAACAGCGGTAAGATCCTTGAGAGTTTTCGCCCCGAAGAACGTTTTCC
AATGATGAGCACTTTTAAAGTTCTGCTATGTGGCGCGGTATTATCCCGTATTGACGCCG
GGCAAGAGCAACTCGGTCGCCGCATACACTATTCTCAGAATGACTTGGTTGAGTACTCA
CCAGTCACAGAAAAGCATCTTACGGATGGCATGACAGTAAGAGAATTATGCAGTGCTGC
CATAACCATGAGTGATAACACTGCGGCCAACTTACTTCTGACAACGATCGGAGGACCGA
AGGAGCTAACCGCTTTTTTGCACAACATGGGGGATCATGTAACTCGCCTTGATCGTTGG
GAACCGGAGCTGAATGAAGCCATACCAAACGACGAGCGTGACACCACGATGCCTGTAG
CAATGGCAACAACGTTGCGCAAACTATTAACTGGCGAACTACTTACTCTAGCTTCCCGG
CAACAATTAATAGACTGGATGGAGGCGGATAAAGTTGCAGGACCACTTCTGCGCTCGG
CCCTTCCGGCTGGCTGGTTTATTGCTGATAAATCTGGAGCCGGTGAGCGTGGATYTCG
CGGTATCATTGCAGCMCTGGGGCCAGATGGTAAGCCCTCCCGTATCGTAGTTATCTAC
CCGACGGGGAGTCAGGCACTWTGGATGAAC

Candidate 2:

GGAGSATTGTTTCATCACCACCGTCACCCAGCAGTACGAGTCCGTCGCTTCACCTCGGT
TGGCAAAGGCAATCGCTGAGGGAGACGAGTCACTATTTCCAGGCATTGAGGAGGCACC
GCTAAACCGTGGCCTCTTGATTCTCGCCCAGATGTCTAGCCAAGGCAATTTCATGAACA
AGGAGTACACTGAGGCTTGTGTGGAGGCGGCTAGGGAGCACAAGGACTTTGTCATGGG
CTTCATCTCCCAGGAGACGCTCAACACGGAAGGCGACGATGCCTTTGTCCACATGACC
CCAGGCTGCCAGCTCCCCCCCGAAGATGAAGACCAGCAGGCGAACGGCACCGTTGGA
GGCGATGGCCAAGGCCAGCAGTACAACACGCCGCATAAGATTATTGGCCTCTGCGGTA
GCGACATTGCCATTGTGGGTAGAGGTATCCTCAAGGCCTCGGACCCCGTAGGTCCAGT
TCACCACCGGCACCTCGATTGCTGGCTGCGGACAGCTCGACATTGCCTGCATCTGCAA
GAACGCCGATTTCCTTGCCCATCAAACTCTCTGCCGCTAGCTATTGATTCGTTTGAATCA
TTCTGTAATCAAGTTACCTAGAGATCATAATATTACAAACATTCCAATAGTTAAGAAACAA
TCCCCTACTCTATTAGTTAAAAATGCTGATAAAGAAAGAGGCAGAACGATACCGGTCAG
CGGCGTGGAAAGCCTACACAGAGAGGCTGCTGCGGTAGAAGGGAGAAAAACAAAAGCA
AGGGGGGGGAGTGCGGAGCTGGGAGAAGCTCCTGTCCAATGCCCGCATCATAGAATG
CAATGTATTTAGGCGAACTCTGTCTTGTTCTTCCTTTTTGTGGTCTAGCTGGGACGTTGC
CCAGCTACGTTGATATCATTGCATGCACAAAGGTGGAGGCGAACTGAACATTCTGATAA
TAATTAATTAAGACGTCAGAATTCTCGAGGCGGCCGCATGTGCGTCTCCCTATAGTGAG
TCGTATTAATTTCGCGGGCGGAACCCCTATTTGTTTATTTTTCTAAATACATTCAAATATG
TATCCGCTCATGAGACAATAACCCTGATAAATGCTTCAATAATATTGAAAAAGGAAGAGT

ATGAGTATTCAACATTTCCGTGTCGCCCTTATTCCCTTTTTTGCGGATTTTGCCTTCCTGT
TTTG

Candidate 3:

GAGSATTGTTTCATCACCACCGTCATTCCGTCTACCCATCTGAGAGTTGGTTTTGGAATA
ATATGAATTTTAGGGTATAACTGATGGCAGTGAAGTAAAGAGGCAGAACGATACCGGTC
AGCGGCGTGGAAAGCCTACACAGAGAGGCTGCTGCGGTAGAAGGGAGAAAAACAAAA
GCAAGGGGGGGGAGTGCGGAGCTGGGAGAAGCTCCTGTCCAATGCCCGCATCATAGA
ATGCAATGTATTTAGGCGAACTCTGTCTTGTTCTTCCTTTTTGTGGTCTAGCTGGGACGT
TGCCCAGCTACGTTGATATCATTGCATGCACAAAGGTGGAGGCGAACTGAACATTATCA
TGGTTTTTACCTCCTGAATTCGGATCCCTCGAGCGATACACACTTCTATAGTGTCACCTA
AATGCGTTTAAACCTTCCTGCAGGTGACGATTACCTAACAATCGGTCGATTCGTTTGATG
TTATGTTTTGTTCTCGCTTTGGTTGGCAGGTTACGGCCAAGTTCGGTAAGAGTGAGAGT
TTTACAGTCAAGTAATGCGTGGCAAGCCAACGTTAAGCTGTTGAGTCGTTTTAAGTGTAA
TTCGGGGCAGAATTGGTAAAGAGAGTCGTGTAAAATATCGAGTTCGCACATCTTGTTGT
CTGATTATTGATTTTTCGCGAAACCATTTAATCATATGACAAGATGTGTGTCCACCTTAAC
TTAATGATTTTTACCAAAATCATTAGGGGATTCATCAGCGCTGAGTGTGTAAATTAATTTT
TATGCCGCAGCGGGCCAGCAATTCTCGTGAATCATCGCTTAAACGGCCTGATTTCTGAA
TAGCTATGCGTAAGCGGGTGTTGTCTAACATTCTGCGTTCCTCTTTATCCTGTCTGAACC
GGCTGCATTAATGAATCGGCCAACGCGCGGGGAGAGGCGGTTTGCGTATTGGGCGCT
CTTCCGCTTCCTCGCTCACTGACTCGCTGCGCTCGGTCGTTCGGCTGCGGCGAGCGGT
ATCAGCTCACTCAAAGGCGGTAATACGGTTATCCACAGAATCAGGGGATAACGCAGGAA
AGAACATGKGAGCAAAAGGCCAGCAAAAGGCCAGGAACCGTAAAAAGGCCCATTGCTG
GCGTTTTCATAGGCTCCGCCCCCTGACRR

Candidate 4:

CATTGTTTCCMTCACCACCGTCAAAGAGGCAGAACGATACCGGTCAGCGGCGTGGAAA
GCCTACACAGAGAGGCTGCTGCGGTAGAAGGGAGAAAAACAAAAGCAAGGGGGGGGA
GTGCGGAGCTGGGAGAAGCTCCTGTCCAATGCCCGCATCATAGAATGCAATGTATTTAG
GCGAACTCTGTCTTGTTCTTCCTTTTTGTGGTCTAGCTGGGACGTTGCCCAGCTACGTT
GATATCATTGCATGCACAAAGGTGGAGGCGAACTGAACATTATCATGGTTTTTACCTCCT
GAATTCGGATCCCTCGAGCGATACACACTTCTATAGTGTCACCTAAATGCGTTTAAACCT
TCCTGCAGGTGACGATTACCTAACAATCGGTCGATTCGTTTGATGTTATGTTTTGTTCTC
GCTTTGGTTGGCAGGTTACGGCCAAGTTCGGTAAGAGTGAGAGTTTTACAGTCAAGTAA
TGCGTGGCAAGCCAACGTTAAGCTGTTGAGTCGTTTTAAGTGTAATTCGGGGCAGAATT
GGTAAAGAGAGTCGTGTAAAATATCGAGTTCGCACATCTTGTTGTCTGATTATTGATTTT
TCGCGAAACCATTTAATCATATGACAAGATGTGTGTCCACCTTAACTTAATGATTTTTACC
AAAATCATTAGGGGATTCATCAGCGCTGAGTGTGTAAATTAATTTTTATGCCGCAGCGG
GCCAGCAATTCTCGTGAATCATCGCTTAAACGGCCTGATTTCTGAATAGCTATGCGTAA
GCGGGTGTTGTCTAACATTCTGCGTTCCTCTTTATCCTGTCTGAACCGGCTGCATTAATG
AATCGGCCAACGCGCGGGGAGAGGCGGTTTGCGTATTGGGCGCTCTTCCGCTTCCTCG
CTCACTGACTCGCTGCGCTCGGTCGTTCGGCTGCGGCGAGCGGTATCAGCTCACTCAA
AGGCGGTAATACGGTTATCCACAGAATCAGGGGATAACGCAGGAAAGAACATGTGAGC
AAAAGGCCAGCAAAAGGCCAGGAACCGTAAAAAGGCCGCATTGCTGGCGTTTTTCCATA

GGCTCCGCCCCCCTGACGAGCATCMCAAAAATCGACGCTCAAGTCAGAGGGGCGAAA
CCCGACAGGACTWTAAAGAWACCAGGCGTTTCCCCCTGGAAGCTCCCTCG
